# Two new species of Tornidae (Caenogastropoda, Rissooidea) from Espírito Santo, Brazil

**DOI:** 10.3897/zookeys.238.3884

**Published:** 2012-11-06

**Authors:** Luiz Ricardo L. Simone

**Affiliations:** 1 Museu de Zoologia da Universidade de São Paulo. Cx. Postal 42494; 04299-970 São Paulo, SP. Brazil

**Keywords:** Tornidae, Caenogastropoda, biodiversity, coastal waters, Espírito Santo, Brazil

## Abstract

Two new species of shallow water Tornidae are found in Espírito Santo state, Brazil, formally described herein. They belong to a complex group of tiny gastropods, in such the taxonomy is very confused. *Cyclostremiscus mohicanus* **sp. n.** is characterized by three well-developed spiral, equidistant carinas, working as base of three series of tall, aligned periostracal rods. *Episcinia itanhuna* **sp. n.** has as single sculpture a series of pustules in periphery, but the periostracum bears three series of peripheral fringes with irregular rods. The new species are compared with the allies, showing a close relation with Caribbean fauna, but possessing worthy differences. These similarities have raised misidentifications.

## Introduction

The Tornidae Sacco, 1896 has also been called Vitrinellidae Bush, 1897. They normally are tiny (~2 mm), discoid gastropods living in coastal shallow waters, usually associated with other organisms, such as algae, worm galleries, etc.

Possibly because of the minute size, the tornids are normally absent in faunal inventories. In collections, the few samples are normally poorly identified, and the draft identification, at least in Brazilian samples, is normally south expansions of North Atlantic or Caribbean species, resulting exceedingly wide distributed species. The same has reflected in the pertinent literature. This wide distribution contrasts with the minute size and the paucispiral protoconch, both normally indicating short or no planktonic phase, and low dispersion. On the other hand, this supposedly wide distribution does not resist to a close look, in such interesting and important differences appear confronting distant collected samples. This paper is just another example. It deals with two species so far identified as species described from Florida and North Carolina, which has been extended to Caribbean. However, some significant details have demonstrated that the Brazilian samples actually belong to different, undescribed species.

The western Atlantic tornid fauna had an important improvement with a recent revision (Rubio et al. 2011), allowing a better analysis of samples. The genus *Cyclostremiscus* Pilsbry & Olsson, 1945 (type species, OD, *Vitrinella panamensis* C.B. Adams, 1852, from Caribbean) has a worldwide, tropical distribution. It is mainly characterized by usually carina-bearing shell, normally with secondary sculpture in intervals of carinas, and a wide, opened umbilicus (Pilsbry and Olsson 1945: 266; Rubio et al. 2011: 83). The genus *Episcynia* Mörch, 1875 (type species, M, *Solarium inornatum* d’Orbigny, 1842, from Caribbean), is restricted to Pacific and Atlantic coasts of Americas. It is mainly characterized by serrate peripheral keel, deep umbilicus, and spiral fringes with periostracal filaments (Rubio et al. 2011: 125). This paper deals with the formal descriptions of two species belonging to these genera, recently collected in sediment sorting by local researchers, as well as confrontation with type specimens.

## Material and methods

The studied samples are only empty shells in all kinds of preservation levels, since specimens with periostracum to eroded shells. They are photographed in multi-focus dissecting microscope and in SEM (Laboratório de Microscopia Eletrônica do Museu de Zoologia da USP).

Abbreviations of institutions are: MNRJ: Museu Nacional da Universidade Federal do Rio de Janeiro; MZSP, Museu de Zoologia da Universidade de São Paulo; USNM, National Museum of Natual History, Smithsonian Institution.

## Systematics

### Genus *Cyclostremiscus* Pilsbry & Olsson, 1945

#### Cyclostremiscus mohicanus sp. n.

urn:lsid:zoobank.org:act:FF9F3D9C-8BF9-4CAB-A236-8C143BA62A6F

http://species-id.net/wiki/Cyclostremiscus_mohicanus

Figs 1–15

##### Types.

**Figures 1–15.**
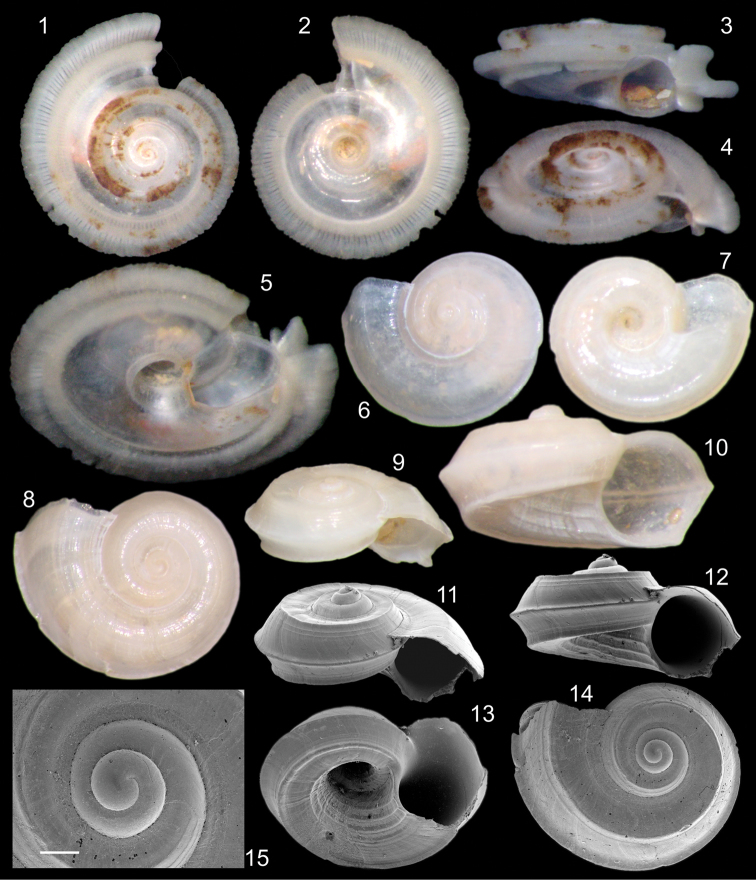
*Cyclostremiscus mohicanus* sp. n. types **1** holotype MZSP 106551 (W 2.8 mm), apical view **2** same, umbilical view **3** same, apertural view **4** same, apertural-slightly apical view **5**, same, apertural-slightly umbilical view **6** paratype MZSP 106552 #1, apical view (W 1.8 mm) **7** same, umbilical view **8** #2, apical view (W 1.7 mm) **9** #1, apertural-slightly apical view **10** same, apertural view **11** #3, SEM, apertural-slightly apical view (W 1.7 mm) **12** same, apertural view **13** same, apertural-slightly umbilical view **14** #4, SEM, apical view (W 1.5 mm) **15** same, detail of apical region, scale 0.1 mm.

Holotype MZSP 106551 (Figs 1–5). Paratypes: 106552, 16 shells from type locality.

##### Type locality.

BRAZIL. **Espírito Santo**; São Mateus, Guriri, 18°47'S, 32°39'W, 3 m depth.

##### Diagnosis.

Shell of about 2.5 mm; almost planispiral. Three carina-like spiral threads somewhat equidistant; superior surface smooth or with scanty spiral cords. Periphery smooth except threads. Peri-umbilical area with string spiral cords. Periostracum with aligned series of tall rods on spiral threads.

##### Description.

**Shell**. Up to ~2.5 mm, discoid; height ~52% of maximum width (Figs 3, 10, 12). Color pure white, weakly translucent (Figs 1, 2, 5, 6). Protoconch of 2 whorls, weakly-turbiform (Figs 4, 10, 11, 15); whorls of rounded profile, suture shallow; surface glossy, smooth; occupying ~10% of shell size; located almost central, weakly dislocated towards right. Transition protoconch-teleoconch unclear (Fig. 15). Spire weakly elevated, with ~45% of shell width; ~15% of shell height. Teleoconch up to 2.5 whorls, uniformly growing weakly planispiral, bearing three somewhat equidistant carinas (Figs 9, 10, 11, 12); peripheral carina as tallest, profile blunt, ~100°, located in middle level of last whorl, inflating ~15% shell width; inferior carina similar to peripheral carina, located midway between this and peri-umbilical slope (Figs 10, 12, 13); superior carina with ~half size as peripheral carina, located midway between this and adjacent suture (Figs 6, 8, 9, 11); between carinas somewhat planar surface, being weakly elevated in carina’s base. Sculpture superior to peripheral carina absent (Figs 11, 14) to series of narrow, low, obsolete spiral cords (Figs 6, 8), varying from zero (surface smooth, Fig. 14) to ~10 (Fig. 8); interspaces between spiral cords ~1/4 their width; sculpture inferior to peripheral carina absent (smooth). Umbilicum widely opened; maximum diameter ~30% shell width; flanking by strong, somewhat planar slope; sculptured from 1 to 5 spiral cords, with interspaces ~3 times their width (Figs 5, 7, 12, 13). Aperture weakly prosocline (Figs 4, 9, 11, 13); rounded, weakly pentangular, i.e., bearing somewhat equidistant blunt angles produced by three carinas and umbilical slope (Figs 10, 12); with ~35% shell width, ~70% of shell height. Callus practically absent, weakly covering adjacent whorl in apertural implantation.

Periostracum (Figs 1–5). Opaque, transparent, color pale beige. Series of tall rods aligned on three carinas; rods of peripheral carina augmenting ~10% shell width (Figs 1, 2), about twice taller than wide, tip rounded, slightly broader than base; rods of superior carina similar to those of peripheral carina, with ~80% of their size (Figs 3, 4); rods of inferior carina also similar to those of peripheral carina, with ~30% their height and ~60% their width (Figs 3, 5). Each rod blade-like, flexible, located close to each other from same carina, forming tall flexible ridge on each carina. Periostracum ridge on three carinas suddenly finishing at apertural level. On aperture, region between ridge of superior carina and insertion of outer lip in adjacent preceding whorl a small region with ridge of peripheral ridge reabsorbed, forming anal notch with ~1/5 of aperture size (Figs 1, 2, 5).

**Measurements** (in mm). Holotype: 2.8 by 0.9; paratype 106552 (Fig. 10): 1.7 by 0.8.

##### Distribution.

Only known from type locality.

##### Habitat

. Sandy bottoms, 3 m depth (no living specimens).

##### Material examined.

Types.

##### Discussion.

*Cyclostremiscus mohicanus* is similar to *Cyclostremiscus beauii* (Fischer, 1857) (Rosenberg et al. 2009; Rubio et al. 2011) from Florida and Caribbean. It differs by the smoother superior surface, lacking so developed spiral cords, the contrary happens in the umbilicum, in such that of *Cyclostremiscus mohicanus* has a series of spiral cords, while *Cyclostremiscus beauii* has only growth lines; the size is also different, as *Cyclostremiscus mohicanus* has about 3 mm, while *Cyclostremiscus beauii* reaches 9-10 mm. *Cyclostremiscus mohicanus* also resembles *Cyclostremiscus pentagonus* (Gabb, 1873), also from Caribbean, it differs by the more developed spiral sculpture in the superior shell surface, by the peri-umbilical spiral sculpture, in being slightly taller (height/width tax= ~52% against ~48% of *Cyclostremiscus pentagonus*), and in having the peripheral carina slightly more elevated. *Cyclostremiscus pentagonus* has been referred as occurring in south Brazil (Rios 2009: 59, in Porto Belo, Santa Catarina; Rubio et al. 2011: 91); as that material was not found, this record is here considered doubtful, but possibly they are of *Cyclostremiscus mohicanus*. Another important difference between *Cyclostremiscus mohicanus* and *Cyclostremiscus pentagonus* is the protoconch, it has ~2 whorls (Fig. 15), while that of *Cyclostremiscus pentagonus* is ~0.5 whorl longer (Rubio et al. 2011, fig. 46F). *Cyclostremiscus mohicanus* is also somewhat similar to *Cyclostremiscus trilix* (Bursh, 1885), sharing the size and the carinas shape; however, it differs from that species in lacking the microtubercles on the protoconch, in having spiral sculpture in surface between suture and superior carina, and in being taller (height/width tax= ~52% against ~42% of *Cyclostremiscus trilix*).

The periostracum bearing expansions are relatively common in living and fresh-died specimens of tornids. However, a periostracum with the *Cyclostremiscus mohicanus* arrangement appears to be novelty. Nothing like that has been found in other congeneric species. *Cyclostremiscus mohicanus* clearly belongs to the “group 1” as defined by Rubio et al. (2011: 84), which encompasses carinate species of the genus, with 2 or 3 peripheral carinas. Possibly all carinas of those species are base of periostracal expansions like those of *Cyclostremiscus mohicanus*.

### Genus *Episcynia* Mörch, 1875

#### Episcynia itanhura sp. n.

urn:lsid:zoobank.org:act:154F48BF-4FB0-44B1-B83A-62C1650CB74E

http://species-id.net/wiki/Episcynia_itanhura

Figs 16–27

Episcinia inornata : Rios 1994: 59 (fig. 221), 2009: 101 (fig. 245) (non d’Orbigny, 1842).
Episcinia inornata form *“multicarinata”*: Rubio et al. 2011: 126 (fig. 68D) (non Dall, 1889).

##### Types. 

**Figures 16–27.**
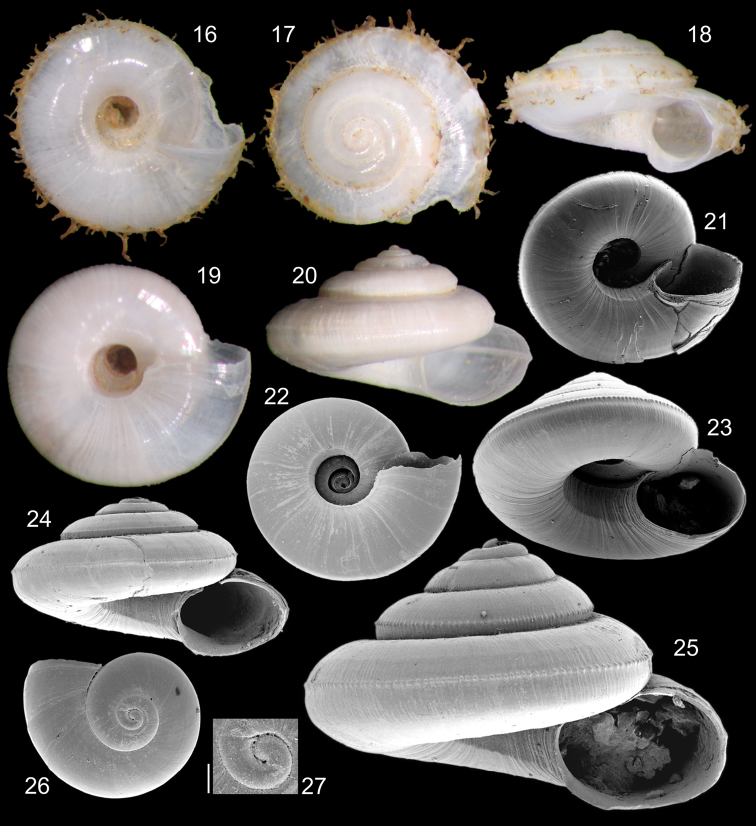
*Episcynia itanhura* sp. n. types **16** holotype MZSP 106553, umbilical view (W 2.0 mm) **17** same, apical view **18** same, apertural view **19** paratype MZSP 106556#1, umbilical view (W 2.2 mm) **20** same, apertural view **21** #2, SEM, umbilical-slightly apertural view (W 2.5 mm) **22** #3, SEM, umbilical view (W 2.1 mm) **23** #4, SEM, apertural-slightly umbilical view (W 2.5 mm) **24** #5, SEM, apertural view (W 2.6 mm) **25** #4, SEM, apertural view **26** #6, SEM, apical view (W 1.0 mm) **27** #6, detail of protoconch (scale 50µm).

Holotype MZSP 106553 (Figs 16-18). Paratypes: 106556, 18 shells, MNRJ, 3 shells, USNM, 3 shells, all from type locality.

##### Type locality.

BRAZIL. **Espírito Santo**; São Mateus, Guriri, 18°47'S, 32°39'W, 3 m depth.

***Diagnosis*.** Shell relatively trochoid. Periostracum with 3 series of peripheral fringes constituted of irregular rods. Surface smooth except for peripheral line of pustules. Peripheral carina wanting (profile rounded).

##### Description.

**Shell**. Up to ~2.5 mm, discoid (Figs 18, 24) to trochiform (Figs 20, 25); height ~57-68% of maximum width (Figs 18, 20, 24, 25). Color white, weakly translucent (Figs 16–20). Protoconch of 1 whorl, ~90 µm, weakly-turbiform (Figs 26, 27); whorl of rounded profile, suture shallow; surface glossy, smooth; occupying ~7% of shell size; located almost central, weakly dislocated towards right (Fig. 17). Transition protoconch-teleoconch clear, orthocline (Figs 26, 27). Spire dome-shaped to elevated, with ~66% of shell width; ~60–64% of shell height. Teleoconch up to 4 whorls, uniformly growing inferiorly; last whorl with rounded profile, lacking peripheral carina. Sculpture practically absent, except for growth lines looking undulations and aligned set of small pustules located just superiorly to suture and in middle level of past whorl periphery; each pustule rounded, separated from neighboring pustules by space equivalent to half its width; 2 to 4 equidistant, very weak spiral cords present in ~50% of specimens located in superior region of each whorl (Figs 20, 25); inferior region of body whorl only with growth lines (Figs 19, 22). Umbilicum widely opened; maximum diameter ~30% shell width; flanking by strong, somewhat planar slope, beating only growth lines; weak angulation marking periphery of umbilicum (Figs 19, 23, 24, 25). Aperture slightly prosocline (Figs 21, 22, 23); elliptical (longer axis vertical) (Figs 24, 25); with ~36% shell width, ~40–43% of shell height. Callus practically absent, weakly covering adjacent whorl in apertural implantation (Figs 21, 23).

Periostracum (Figs 16–18). Opaque, transparent, color yellowish beige (Figs 16–18). Three series of rods running on periphery (Fig. 18); central fringe running on pustule lines; other 2 fringes running above and below central fringe, distance between fringes equivalent to ~1/3 of whorl height; periostracum rods located on fringes not uniformly distributed and sized; longer rods extending ~10% shell width, weakly coiled, distantly separated from neighbor rods.

**Measurements** (in mm). Holotype (Fig. 18): 2.0 by 1.1; paratypes MZSP 106556 (Fig. 20)#1: 2.2 by 1.3; (Fig. 24)#5: 2.6 by 1.5; (Fig. 25)#4: 2.5 by 1.6.

##### Distribution.

Brazil, from Bahia to São Paulo.

##### Habitat.

Sandy bottoms, ~3 m depth (no living specimens collected).

##### Material examined.

Types. Additional material: MZSP 106571, 90 shells from type locality. BRAZIL. **Bahia**; Salvador, Itapoã beach, MZSP 53439, 1 shell (C.M. Cunha col.). **Rio de Janeiro**; Cabo Frio, MZSP 57159, 2 shells (17/ii/1970). **São Paulo**; off Ubatuba, MZSP 88431, 1 shell (Veliger II, sta. PI-15; 21/i/1986); 23°33'978"S, 45°09'821"W, 12.3 m depth, MZSP 42087, 1 shell (Biota sta. 145i; 16/iv/2002). N.B. These samples are not part of the type material because of low quality of the shell preservation.

##### Etymology.

The specific epithet is derived from the Paritintin word Itanhura’mbi – chain used as necklace (Betts 1981), an allusion to the peripheral ornamentation of the shell.

##### Discussion.

*Episcynia itanhura* is similar to *Episcynia inornata* (d’Orbigny, 1842), from Florida and Caribbean, differing in lacking so developed peripheral carina, the profile of each whorl is rounded while that of *Episcynia inornata* is bluntly pointed (Rubio et al. 2011, Figs 68B, 69); *Episcynia itanhura* has a more developed axial undulation, almost sculpture (e.g., Figs 20, 25), this is rare in *Episcynia inornata*, in such the surface is smoother and glossy. The size of the protoconch of *Episcynia inornata* has been referred as 190 µm (Rubio et al. 2011), while that of *Episcynia itanhura* is about half that size (~90 µm, Figs 26, 27). The periostracum rods are organized in 3 fringes in *Episcynia itanhura* (Fig. 18), while a single pair is found in *Episcynia inornata* (Andrews 1971: 68; Abbott 1974: 86). The 3 fringes are also found in *Episcynia multicarinata* (Dall 1889), from North Carolina to north Caribbean, *Episcynia itanhura* differs in having periostracum rods more sparsely and less uniformly developed, by the deeper suture, and by the straighter profile of spire. The differences between Caribbean and Brazilian specimens have been pointed in literature (Rubio et al. 2011, fig. 68D), however, the differences obviously did not influenced the specific separation. Nevertheless, some doubt still remains in relation to the specimen figures in that paper (Rubio et al. 2011), because that illustrated specimen has whorls with almost squared profile, possibly it belong to another undescribed specimen. No specimen with such features has been examined herein. Additionally, there is some uncertainties respect to the possible synonymy between *Episcynia inornata* and *Episcynia multicarinata* (Dall 1889), described from North Carolina. Dall (1889: 392-393) clearly stated a specimen with four to five carinas per whorl. This feature is not found in *Episcynia inornata* or allied species; this can demonstrate a valid entity. Moreover, Dall still described a more richness of sculpture, a lack of periostracal fringe in peripheral carina, and color yellow, whose can be extra indicative of specific differentiation. Despite further studies are necessary to clarify the question, *Episcynia itanhura* cannot be confused with *Episcynia multicarinata*.

## Supplementary Material

XML Treatment for Cyclostremiscus mohicanus

XML Treatment for Episcynia itanhura

## References

[B1] Abbott RT (1974) American Seashells, second edition. Van Nostrand Reinhold Company. New York, 663 pp., 240pls.

[B2] Altena COvR (1966) Vitrinellidae (marine Mollusca Gastropoda) from Holocene deposits in Surinam (Dutch Guiana). Zoologische Mededelingen 41 (16): 233-241

[B3] Andrews J (1971) Sea shells of the Texas coast. University of Texas Press. Austin, 298 pp.

[B4] Betts LV (1981) Dicionário Paritintin-Portugues. Sociedade Internacional de Lingüística. Cuiabá, 231 pp.

[B5] Dall WH (1889) Reports on the results of dredgings, under the supervision of Alexander Agassiz, in the Gulf of Mexico (1877–78) and in the Caribbean Sea (1879–80), by the U. S. Coast Survey Steamer ‘Blake’. Bulletin of the Museum of Comparative Zoology 18: 1–492, pls. 10–40.

[B6] Pilsbry HA Olsson AA (1945) Vitrinellidae and similar gastropods of the Panamic Province. Part I. Proceedings of the Academy of Natural Sciences of Philadelphia 97: 249-278

[B7] Rios EC (1994) Seashells of Brazil. Second edition. Fundação Universidade do Rio Grande. Rio Grande, 368 pp., 113 pls.

[B8] Rios EC (2009) Compendium of Brazilian seashells. Universidade Federal do Rio Grande. Rio Grande, 668 pp.

[B9] Rosenberg G Moretzsohn F García EF (2009) Gastropoda (Mollusca) of the Gulf of Mexico. In: Felder DL Camp DK (Eds). Gulf of Mexico – Origins, Waters, and Biota.Biodiversity. Texas A&M Press, College Station. Texas: 579-699

[B10] Rubio F Fernández-Garcés R Rolán E (2011) The family Tornidae (Gastropoda, Rissooidea) in the Caribbean and neighboring areas. Iberus 29 (2): 1-230

